# Comprehensive characterization of toxicity of fermentative metabolites on microbial growth

**DOI:** 10.1186/s13068-017-0952-4

**Published:** 2017-11-30

**Authors:** Brandon Wilbanks, Cong T. Trinh

**Affiliations:** 1 Department of Chemical and Biomolecular Engineering, University of Tennessee, 1512 Middle Drive, Knoxville, TN 37996 USA; 2 Bioenergy Science Center, Oak Ridge National Laboratory, Oak Ridge, USA

**Keywords:** *Escherichia coli*, Toxicity, Alcohols, Carboxylic acids, Esters, Growth inhibition, Energy density, Partition coefficient, Hydrophobicity, Total surface area

## Abstract

**Background:**

Volatile carboxylic acids, alcohols, and esters are natural fermentative products, typically derived from anaerobic digestion. These metabolites have important functional roles to regulate cellular metabolisms and broad use as food supplements, flavors and fragrances, solvents, and fuels. Comprehensive characterization of toxic effects of these metabolites on microbial growth under similar conditions is very limited.

**Results:**

We characterized a comprehensive list of thirty-two short-chain carboxylic acids, alcohols, and esters on microbial growth of *Escherichia coli* MG1655 under anaerobic conditions. We analyzed toxic effects of these metabolites on *E. coli* health, quantified by growth rate and cell mass, as a function of metabolite types, concentrations, and physiochemical properties including carbon number, chemical functional group, chain branching feature, energy density, total surface area, and hydrophobicity. Strain characterization revealed that these metabolites exert distinct toxic effects on *E. coli* health. We found that higher concentrations and/or carbon numbers of metabolites cause more severe growth inhibition. For the same carbon numbers and metabolite concentrations, we discovered that branched chain metabolites are less toxic than the linear chain ones. Remarkably, shorter alkyl esters (e.g., ethyl butyrate) appear less toxic than longer alkyl esters (e.g., butyl acetate). Regardless of metabolites, hydrophobicity of a metabolite, governed by its physiochemical properties, strongly correlates with the metabolite’s toxic effect on *E. coli* health.

**Conclusions:**

Short-chain alcohols, acids, and esters exhibit distinctive toxic effects on *E. coli* health. Hydrophobicity is a quantitative predictor to evaluate the toxic effect of a metabolite. This study sheds light on degrees of toxicity of fermentative metabolites on microbial health and further helps in the selection of desirable metabolites and hosts for industrial fermentation to overproduce them.

**Electronic supplementary material:**

The online version of this article (10.1186/s13068-017-0952-4) contains supplementary material, which is available to authorized users.

## Background

During anaerobic digestion of organic matters, organisms naturally produce volatile organic acids and alcohols to balance cellular redox states. These molecules, along with esters generated from condensation of alcohols and acids, are of particular interest for not only fundamentally studying their functional roles to regulate cellular metabolisms and microbiomes [1] but also harnessing them as food supplements, natural flavors and fragrances, solvents, and fuels [2].

A diverse class of microbes can naturally produce these volatile metabolites, some being harnessed for industrial-scale production. For instance, *Escherichia coli,* a facultative, gram-negative bacterium found in the lower intestine of animals, is widely used as an industrial workhorse microorganism for biocatalysis. *E. coli* possesses a native mixed acid fermentative metabolism that has been metabolically engineered to produce many fermentative metabolites, including alcohols (e.g., ethanol [3, 4], isopropanol [5], butanol [6], isobutanol [7], pentanol [8], and hexanol [9]), diols (e.g., 1,3-propanediol [10] and 1,4-butanediol [11]), acids (e.g., pyruvate [12], lactate [13], and short–medium-chain carboxylic acids [14]), diacids (e.g., succinate [15] and adipate [16]), and esters (e.g., acetate esters [17], propionate esters [18, 19], butyrate esters [18–20], pentanoate esters [18, 19], and hexanoate esters [18, 19]).

Fermentative metabolites, however, can become inhibitory to microbial growth by directly interfering with cell membrane and/or intracellular processes [21–29]. Currently, data on toxic effects of a comprehensive set of fermentative metabolites on microbial growth under similar growth conditions are very limited. Availability of these data can help identify and better understand most toxic metabolites to microbes during fermentation. It also provides design criteria for selecting desirable metabolites and microbes for industrial production as well as guiding effective engineering strategies to alleviate toxicity. For instance, various engineering approaches have been implemented to enhance microbial tolerance against some fermentative metabolites including increasing the ratio of saturated and unsaturated fatty acid compositions [30], raising the average chain length of fatty acid moieties in cell membrane [31], enhancing the ratio of trans- and cis-unsaturated fatty acids of cell membrane [32], and expressing efflux pumps [33] or chaperones [34]. Genome and evolutionary engineering have also been explored to enhance tolerance [24, 35–37].

In this study, we characterized the toxic effects of a comprehensive set of thirty-two fermentative metabolites including eight carboxylic acids, eight alcohols, and sixteen esters on *E. coli* health. We analyzed the toxic effects of these metabolites as a function of metabolite types, concentrations, and physiochemical properties including carbon number, chemical functional group, chain branching feature, energy density, total surface area, and hydrophobicity.

## Results and discussion

To study the toxic effects of fermentative metabolites on *E. coli* health, growth kinetics were generated for each metabolite using industrially relevant concentrations (0, 2.5, 5.0, and 7.5 g/L) and additional concentrations as needed for certain metabolites. Both growth rate and OD during the first 24 h period were extracted to evaluate *E. coli* health. For the reference growth condition without an exogenously added chemical, wildtype *E. coli* MG1655 grew at a rate of 0.61 ± 0.03 1/h and an OD of 1.40 ± 0.06 (Additional file 1: Figures S1–S3).

### Toxic effects of alcohols

**Fig. 1:**
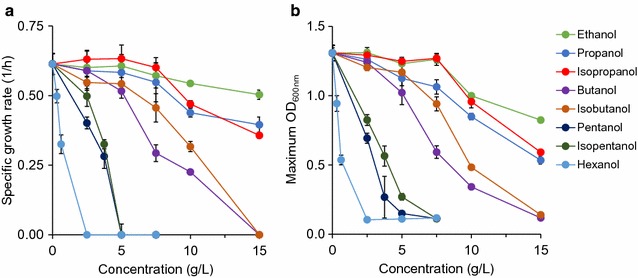
Toxic effects of alcohols on *E. coli* health based on **a** specific growth rate and **b** maximum OD

The first alcohol of interest, ethanol, was found to be essentially non-toxic up to 7.5 g/L (Additional file 1: Figure S1A). At 10 g/L ethanol, specific growth rate and OD decreased by only 12% and 25% each as compared to the reference (without supplementation of toxins) (Fig. 1). At the highest measured concentration of 15 g/L, growth rate was further reduced by only 18%, but OD was nearly 40% lower at 0.82 ± 0.01. This trend of limited growth inhibition by ethanol is consistent with a previous report, where the growth rate of *E. coli* was reduced 25% in a complex medium containing 20 g/L ethanol [38].

Propanol toxicity at concentrations of up to 7.5 g/L was similar to that of ethanol, but at 15 g/L it was more toxic (Additional file 1: Figure S1B). Specific growth rate was 0.40 ± 0.03 1/h (nearly 50% lower than the reference) and OD was 0.53 ± 0.03 (~ 60% lower than the reference) (Fig. 1). Isopropanol toxicity exhibited relatively similar trends like propanol toxicity with slightly higher growth and OD at most concentrations tested (Fig. 1, Additional file 1: Figure S1C).

Butanol was the first alcohol to display strong toxic effects before 10 g/L (Additional file 1: Figure S1D). At 7.5 g/L, growth rate (0.29 ± 0.03 1/h) and OD (0.50 ± 0.05) were reduced more than 50% as compared to the reference (Fig. 1). Growth was entirely inhibited in butanol at 15 g/L. Our data presented for butanol toxicity are consistent with a previous study reporting that growth of *E. coli* DH5α in YPD medium was reduced by 80% in 1% v/v (~ 8.1 g/L) butanol and stopped at 2% v/v (~ 16.2 g/L) [39]. Isobutanol was less toxic than butanol at all concentrations, with the exception of 15 g/L, where no growth was observed for both compounds (Additional file 1: Figure S1E). At 7.5 g/L, isobutanol was less inhibitory than butanol for *E. coli* growth, with higher specific growth rate and OD by approximately 25% (Fig. 1). Findings of isobutanol toxicity presented here are consistent with Atsumi et al.’s report [24]. The difference in toxic effects of isobutanol and butanol is consistent with the data from Huffer et al.’s report [25]. Remarkably, based on Huffer et al.’s data, microbial health is less inhibited in isobutanol than butanol not only for *E. coli* but also for some other bacterial, eukaryotic, and archaeal species.

For pentanol and isopentanol, no growth was observed at any studied concentrations above 5 g/L (Additional file 1: Figures S1F, G). Pentanol terminated all growth at 5 g/L, and at 3.75 g/L specific growth rate was just 0.28 ± 0.04 1/h (Fig. 1, Additional file 1: Figure S1F). Unlike pentanol, isopentanol at 5 g/L allowed for growth, with a reduced specific growth rate of 0.20 ± 0.04 1/h and an OD of 0.27 ± 0.02 (Fig. 1, Additional file 1: Figure S1G). At 2.5 g/L, isopentanol suppressed specific growth rate and OD, respectively, by 12 and 8% less than did pentanol.

Hexanol was the most toxic among alcohols used in this study. It eliminated all growth at only 2.5 g/L. A far reduced concentration of 0.625 g/L still cut growth rate by over 45% and OD by nearly 60% as compared to the reference (Fig. 1, Additional file 1: Figure S1H).

Overall, alcohols are toxic to microbial growth, and degrees of toxicity depend on alcohol types and concentrations. Increasing alcohol concentrations decrease both specific growth rate and OD. Shorter chain length alcohols (ethanol, propanol, isopropanol) require higher concentrations to impact growth.

### Toxic effects of carboxylic acids

**Fig. 2:**
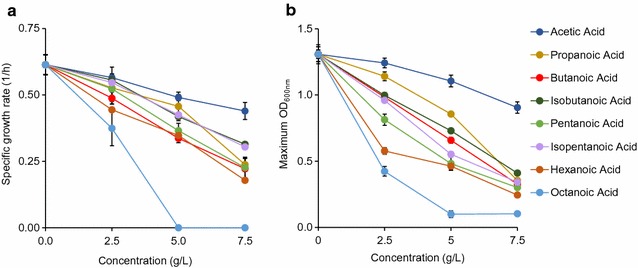
Toxic effects of acids on *E. coli* health based on **a** specific growth rate and **b** maximum OD

Acetic acid was marginally toxic up to 7.5 g/L, at which growth rate (0.44 ± 0.03 1/h) and OD (0.91 ± 0.01) were each reduced by ~ 20% compared to the reference (Fig. 2, Additional file 1: Figure S2A). Propionic acid at an identical concentration was found to be much more toxic than acetic acid, with specific growth rate (0.24 ± 0.03 1/h) and OD (0.35 ± 0.014) being decreased ~ 60 and ~ 75%, respectively (Fig. 2, Additional file 1: Figure S2B).

Butanoic acid at 7.5 g/L was seen to be slightly more inhibitive of growth rate and OD than propionic acid, whereas concentrations of 2.5 and 5 g/L appeared similarly toxic like propionic acid (Fig. 2, Additional file 1: Figures S2C). Isobutanoic acid was found to be less toxic than butanoic acid, following the chain branching trend seen in alcohols (Fig. 2, Additional file 1: Figure S2D). At 2.5, 5.0, and 7.5 g/L, cells grew 6, 5, and 15% faster in isobutanoic acid than butanoic acid.

The pair of pentanoic and isopentanoic acids was also used. At each concentration, isopentanoic acid was less toxic than pentanoic acid. Pentanoic and isopentanoic acids sustained growth at 7.5 g/L to ODs of 0.30 ± 0.05 and 0.34 ± 0.02, and specific growth rates reached 0.23 ± 0.04 and 0.30 ± 0.02 1/h, respectively (Fig. 2, Additional file 1: Figures S2E, F).

The next acid studied was hexanoic acid. Growth with this compound was sustained at 7.5 g/L, but specific growth rate was reduced by > 70% and OD just reached 0.24 ± 0.03 (Fig. 2, Additional file 1: Figure S2G). Octanoic acid was even more toxic, eliminating all growth at 5 g/L (Fig. 2, Additional file 1: Figure S2H). At 2.5 g/L, growth rate (0.37 ± 0.06 1/h) and OD (0.43 ± 0.02) were decreased by about 40 and 65% as compared to the reference, respectively. Octanoic acid was the most toxic organic acid studied here and was the only acid that prevented all growth above 2.5 g/L.

Like alcohols, acid toxicity on microbial growth depends on exposed concentrations and acid chain length. Increasing acid concentrations enhance toxicity for all compounds, reducing growth rates and cell concentrations. Longer chain acids cause severe growth inhibition.

### Toxic effects of esters

Cells are capable of producing a combinatorial library of esters by condensing organic acids and alcohols [18–20]. In this study, we investigated the toxic effects of a comprehensive list of 16 common short-chain esters on *E. coli* health. For comparison, we classified these esters into 3 categories: ethyl esters, propyl esters, and butyl esters.

#### Ethyl esters

**Fig. 3:**
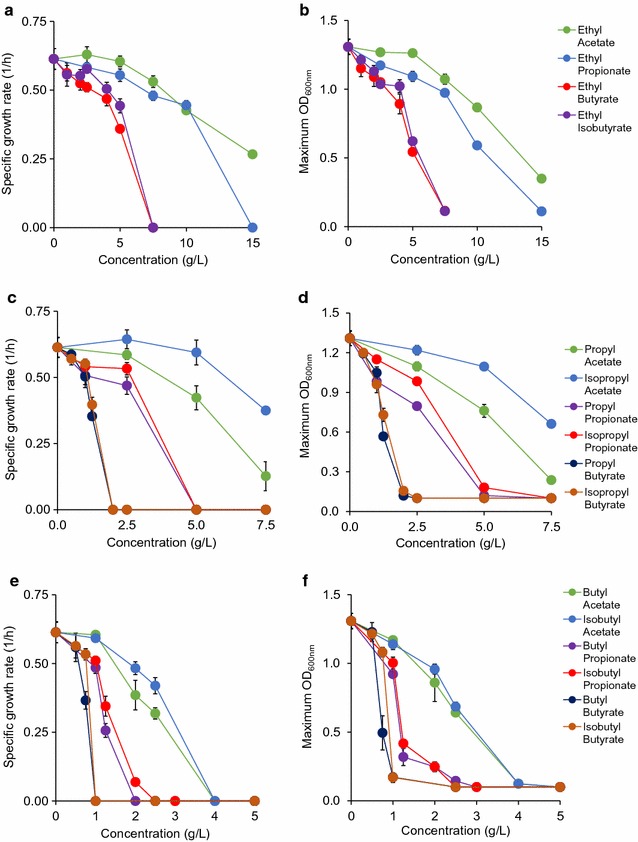
Toxic effects of esters on *E. coli* health based on specific growth rate and maximum OD for **a**, **b** ethyl esters, **c**, **d** (iso)propyl esters, and **e**, **f** (iso)butyl esters

Ethyl acetate was not strongly toxic until concentrations of 10 g/L or greater (Additional file 1: Figure S3A). At 10 and 15 g/L, the specific growth rates observed were reduced to 0.42 ± 0.01 1/h and 0.27 ± 0.01 1/h, respectively. ODs followed a similar trend, being reduced to 0.87 ± 0.03 at 10 g/L and 0.35 ± 0.03 at 15 g/L (Fig. 3). Ethyl propionate was more toxic than ethyl acetate at identical concentrations (Additional file 1: Figure S3B). At 10 g/L, specific growth rates between growth in ethyl acetate and ethyl propionate were not different, but OD was more than 20% lower in ethyl propionate than in ethyl acetate (Fig. 3). No growth occurred with the addition of 15 g/L ethyl propionate, making ethyl acetate the only ester that allowed for any growth at 15 g/L (Additional file 1: Figure S3).

Ethyl butyrate was the most toxic among the characterized ethyl esters, with a specific growth rate of 0.36 ± 0.01 1/h and an OD of 0.54 ± 0.02 at 5 g/L (Fig. 3, Additional file 1: Figure S3C). The toxic effect of ethyl butyrate was still noteworthy at 5 g/L, slowing growth rate by over 25% and lowering OD by over 40% as compared to the reference. The branched chain isomer of ethyl butyrate, ethyl isobutyrate, was also studied (Additional file 1: Figure S3D). It was less toxic than ethyl butyrate at all concentrations, most notably at 5 g/L, where observed growth rate was approximately 20% higher than the growth rate with ethyl butyrate (Fig. 3). Cultures with 7.5 g/L of both ethyl butyrate and ethyl isobutyrate were unable to grow (Additional file 1: Figures S3C, D).

#### Propyl and isopropyl esters

Both propyl acetate and isopropyl acetate inhibited growth at 7.5 g/L, but isopropyl acetate was far less toxic (Additional file 1: Figures S3E, H). Cultures containing propyl acetate at 7.5 g/L reached an OD of 0.24 ± 0.02, doubling only once in 24 h of characterization. However, the cell culture exposed to isopropyl acetate at 7.5 g/L displayed a higher OD than the cell culture exposed to propyl acetate by threefold (Fig. 3). Cells (0.38 ± 0.37 1/h) also grew 3.5 times faster in isopropyl acetate than propyl acetate at this concentration.

The addition of propyl propionate at any concentration 5 g/L or higher prevented all growth (Additional file 1: Figure S3F). A strong toxic effect was seen with the addition of 2.5 g/L of the compound, reducing both specific growth rate (0.47 ± 0.023 1/h) and OD (0.80 ± 0.02) by ~ 25 and ~ 40% as compared to the reference, respectively (Fig. 3). On the other hand, cultures exposed to 2.5 g/L isopropyl propionate displayed much healthier growth (Fig. 3, Additional file 1: Figure S3I), with a specific growth rate of 0.55 ± 0.03 (1/h) and an OD of 0.98 ± 0.02. Like propyl propionate, no growth occurred in cultures at 5 g/L isopropyl propionate.

The final pair of propyl esters characterized here was propyl butyrate and isopropyl butyrate. Both compounds prevented any growth from occurring at 2 g/L, but growth was sustained at concentrations of 1.25 g/L or lower (Additional file 1: Figures S3G, J). Propyl butyrate at 1.25 g/L decreased specific growth rate (0.35 ± 0.34 1/h) and OD (0.57 ± 0.03) by about twofold. Isopropyl butyrate was less toxic, with 7% higher growth rate and 15% higher OD than propyl butyrate at this concentration (Fig. 3).

#### Butyl and isobutyl esters

The addition of butyl acetate reduced both specific growth rate and OD by half at a concentration of 2.5 g/L (Fig. 2, Additional file 1: Figure S3K), while all previously discussed acetate esters (ethyl acetate, propyl acetate, isopropyl acetate) showed no toxic effects at 2.5 g/L or less. No growth was observed at any concentrations of butyl acetate higher than 4 g/L. Isobutyl acetate was less toxic than butyl acetate where cells (0.42 ± 0.03 1/h) grew 15% faster at 2.5 g/L and displayed a 3% increase in OD (0.68 ± 0.03 1/h) (Fig. 3, Additional file 1: Figure S3N). Like butyl acetate, cells exposed to isobutyl acetate at concentrations higher than 4 g/L failed to grow.

Butyl propionate was far more toxic than butyl acetate (Fig. 3, Additional file 1: Figure S3L). Unlike butyl and isobutyl acetates, butyl propionate with a concentration greater than 2 g/L prevented growth. Growth at 1.25 g/L of this compound was marginal with specific growth rate being decreased by more than 60%. The toxic effects were even observed at just 1 g/L, where specific growth rate (0.49 ± 0.02) dropped by 20%. Isobutyl propionate was slightly less toxic, allowing for growth at 2 g/L, but specific growth rate and OD were each no more than 20% of that of the reference (Fig. 3, Additional file 1: Figure S3O).

The final esters of interest were the pair of butyl butyrate and isobutyl butyrate. Butyl butyrate was the most toxic compound in this work, prohibiting all growth at any concentrations of 1 g/L or higher (Fig. 3, Additional file 1: Figure S3M). At just 0.75 g/L, specific growth rate was reduced to 0.37 ± 0.03 1/h (60% of the reference) and OD to 0.49 ± 0.14 (~ 35% of the reference). In comparison, isobutyl butyrate limited growth by 30% less (Fig. 3, Additional file 1: Figure S3P), displaying a growth rate of 0.53 ± 0.02 (1/h) at the same concentration. OD was over twofold higher with this compound than with butyl butyrate. Growth at concentrations of 1 g/L of both compounds was prevented.

Like alcohols and acids, we observed a similar trend of toxicity as a function of ester types and concentrations. Increasing ester concentrations increase toxicity for all compounds and shorter chain esters exhibit less toxic effects on microbial growth.

There was a strong linear correlation (*R* ^2^ > 0.94) between growth rates and cell mass when *E. coli* are exposed to alcohols, acids, and esters (Additional file 1: Figure S4). Therefore, *E. coli* health can be evaluated based on growth rate and cell mass under all conditions investigated.

### Linking physiochemical properties of metabolites and toxic effects

#### Carbon number

**Fig. 4:**
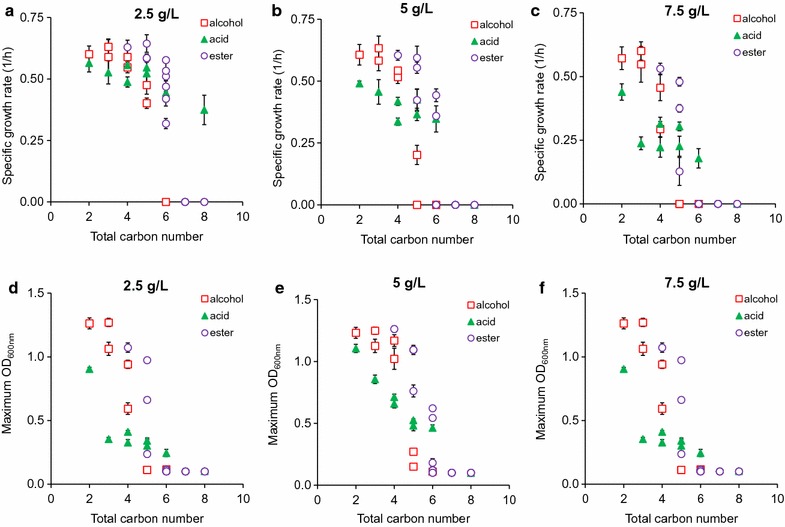
Correlation between the carbon number of a metabolite and its toxic effect on *E. coli* health based on **a**–**c** specific growth rate and **d–f** maximum OD at various initial concentrations of alcohols, acids, and esters in the media

To compare toxic effects of metabolites within and across chemical classes, we first used the total carbon number of a metabolite as a basis. Regardless of chemical types and concentrations, metabolites containing higher carbon numbers were more inhibitory to *E. coli* health, reducing both growth rate and cell mass (Fig. 4). Toxic effects of these metabolites were likely caused by membrane disruption as seen in some acids and alcohols [25, 27, 40, 41], ionic liquids [42], and surfactants [43]. As the carbon number of a metabolite increases, this metabolite becomes more soluble in the cell’s lipid membrane and less so in aqueous media. This interference likely results in extensive adjustment of cell morphology, primarily cell elongation due to change in membrane fluidity, which is a well-known indicator of high-stress environment and damaged membrane [44]. Although the correlation between carbon number and toxic effect of a metabolite was prevalent, the strength of this correlation varied among metabolites within and across metabolite classes (Fig. 4). Therefore, carbon number is not an accurate indicator to evaluate the toxic effect of a metabolite.

#### Chemical functional group

Short-chain alcohols, acids, and esters can exhibit distinct toxic effects on *E. coli* health. Our results showed that acids inhibited growth more strongly than or similarly to alcohols and then esters, for C2–C4 chemicals; however, alcohols became more toxic than organic acids or esters, for ≥ C5 chemicals (Fig. 4). The trend cannot be simply explained alone by the total carbon number of a molecule, but must take into account the chemical functional groups such as the relative polarity of a hydroxyl or a carboxyl group. For example, pentanol and pentanoic acid each have the same carbon number, but pentanol (1.79 D, in debyes) is a less polar pentanoic acid (2.29 D) (Additional file 2: File S2). The higher polarity of pentanoic acid likely makes it less membrane soluble than pentanol at identical concentrations, and hence less toxic on microbial growth. Indeed, our data showed that cells grew faster in pentanoic acid (0.52 ± 0.05 1/h) than in pentanol (0.40 ± 0.02 1/h) at 2.5 g/L and yielded higher cell mass (OD = 0.81 ± 0.02 in pentanoic acid versus 0.69 ± 0.04 in pentanol). Another factor that could potentially contribute to the difference in toxicity of alcohols and acids is steric effect. The larger carboxyl group on organic acids could physically hinder the acid’s ability to enter the membrane, whereas the smaller hydroxyl group could present less resistance.

#### Chain branching

For the same carbon number and chemical class, chain branching can cause different toxic effects on microbial growth. Our result showed that branched chain isomers of each metabolite are less toxic to microbial growth across all chemical classes (Fig. 4 and Additional file 1: Figures S5–S7). This trend can be clearly seen when cells were exposed to C5 alcohols, esters, and acids. At 2.5 g/L exposure, for instance, cells grew ~ 18% faster in isopentanol (0.48 ± 0.04 1/h) than pentanol (0.40 ± 0.02 1/h), 5% faster in isopentanoic acid (0.56 ± 0.02 1/h) than pentanoic acid (0.55 ± 0.05 1/h), and 10% faster in isopropyl acetate (0.64 ± 0.04 1/h) than propyl acetate (0.59 ± 0.02 1/h). The reduced toxic effects of chain branching can be explained by the impact of membrane solubility. Branched chain isomers are less membrane soluble than their corresponding straight chain isomer at any given chain length due to decreased hydrophobicity [45] and hence become less toxic to microbial growth.

#### Ester moieties

Each ester is composed of one alcohol moiety and one acid moiety. Esters containing identical carbon number but different moieties can exert distinct toxic effects on *E. coli* health. For instance, the difference between ethyl butyrate and butyl acetate is that ethyl butyrate is composed of ethanol and butyric acid moieties, while butyl acetate contains butanol and acetic acid moieties. At 2.5 g/L, cells grew ~ 40% slower in butyl acetate (0.3186 ± 0.0207 1/h) than in ethyl butyrate (0.51 ± 0.02 1/h) and also yielded ~ 40% lower cell mass in butyl acetate and ethyl butyrate (Fig. 3, Additional file 1: Figure S3). The same trend was consistently observed in many other ester pairs of the same total carbon number. One explanation for this phenomenon is that an ester (e.g., *P*_butylacetate_ = 69.18, Additional file 2: File S2) with a longer chain alcohol moiety is more hydrophobic and hence more toxic than an ester (e.g., *P*_ethylbutyrate_ = 63.10) with a shorter chain alcohol moiety. The other explanation is based on the extent of ester hydrolysis, which remains to be proven experimentally. A fraction of esters can be hydrolyzed into alcohol and acid moieties in aqueous media; hence, an ester that releases a longer chain alcohol is more toxic.

#### Acid dissociation

For higher carbon numbers, acids appeared less toxic than esters (Fig. 4). For instance, at 7.5 g/L and a total carbon number of 6, cells were still able to grow in acids (hexanoic acid, pKa = 4.6) but neither in alcohols (hexanol) nor in esters (ethyl butyrate, butyl acetate, propyl propionate, isopropyl propionate). One possible explanation for this phenotype is acid dissociation that enables it to exist as a monoprotic acid and a conjugate base. The degree of dissociation depends on pKa of a metabolite and pH. In our experiments, the fraction of conjugate base dominated because the initial pH of media was adjusted to 7. Since the conjugate base is more hydrophilic than the monoprotic acid, it is less membrane soluble and hence less toxic.

#### Energy density

Energy density is one of the most industrially important physical properties of a compound, especially for liquid fuel applications. Among the classes of metabolites investigated in this study, alcohols have the highest energy densities followed by esters and acids with the same carbon numbers primarily because alcohols are least oxygenated (Fig. 6a, Additional file 2: File S2). As the carbon number of a molecule increases, this molecule not only becomes more toxic (Fig. 4) but also has more energy density (Fig. 6a). Thus, it can be predicted that a molecule with a higher energy density is likely more toxic to microbial growth.

#### Hydrophobicity

To better capture the toxic effects of metabolites within and across different classes of metabolites, we further examined metabolite hydrophobicity as a basis for toxicity. We used partition coefficients to determine and quantitatively compare hydrophobicity of metabolites. We found that for the same carbon number, chemicals have different partition coefficients, depending on chemical functional groups and chain branching (Additional file 2: File S2). For instance, partition coefficients of pentanol, isopentanol, pentanoic acid, isopentanoic acid, ethyl propionate, and propyl acetate are 29.5, 15.1, 21.9, 16.2, 20.9, and 19.1, respectively. Experimentally, we observed that pentanol was more toxic than isopentanol, pentanoic acid was more toxic than isopentanoic acid, and ethyl propionate was more toxic than propyl acetate; pentanol with the highest partition coefficient was the most toxic molecule among the C5 chemicals investigated in this study.

**Fig. 5:**
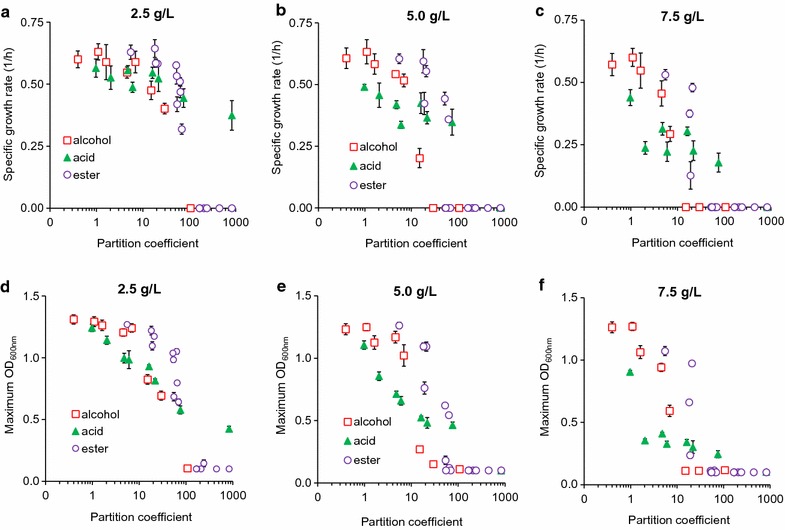
Correlation between the hydrophobicity (i.e., partition coefficient) of a metabolite and its toxic effect on *E. coli* health based on **a**–**c** specific growth rate and **d**–**f** maximum OD at various initial concentrations of alcohols, acids, and esters in the media

Regardless of metabolite types and concentrations, a correlation existed between hydrophobicity of a metabolite and its toxic effect on microbial growth (Fig. 5). As partition coefficients increased, negative effects on specific growth rates and ODs also increased. The negative effects became amplified when cells were exposed to higher chemical concentrations. Among different classes of metabolites examined in this study, alcohols became more toxic than acids and esters for higher partition coefficients (logP ≥ 10). In contrast, esters appeared to be least toxic among different classes of metabolites for lower partition coefficients (logP ≤ 10). We observed that all compounds that prevented growth at concentrations greater than 2.5 g/L have a partition coefficient at least ~ 250 times greater than that of ethanol. Every branched chain isomer in this work was shown to be less toxic than the associated straight chain isomer (Additional file 1: Figures S5–7, Additional file 2: File S2), and in each case the branched chain has a lower partition coefficient than the straight chain compound. Hydrophobicity can differentiate the toxic effects between not only linear and branched molecules but also two esters having an identical carbon number but different alcohol and acid moieties.

**Fig. 6:**
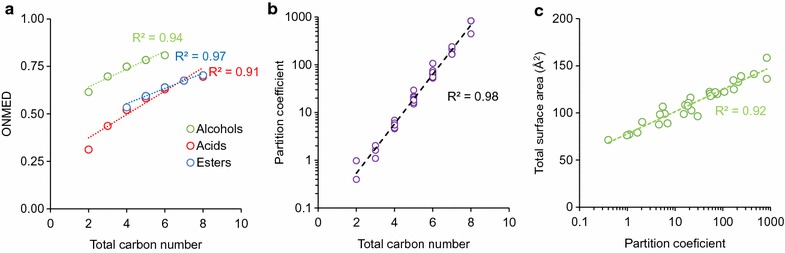
Correlations between **a** octane normalized mass energy density (ONMED) and carbon number, **b** partition coefficient and carbon number, and **c** total surface area and partition coefficient based on physiochemical properties of short-chain alcohols, acids, and esters

Hydrophobicity of a metabolite and its effect on microbial growth can be explained by hydrophobic interaction between the metabolite and cell membrane. As partition coefficients increase, metabolites likely become more membrane soluble and disrupt lipid membranes, which enhance degrees of toxicity and alter cell morphology more severely [46–48]. Remarkably, we found that there existed strong correlations among partition coefficients, carbon numbers, and total surface areas of metabolites (Fig. 6b, c). Different from the total carbon numbers and total surface areas, hydrophobicity can better predict toxicity differences among molecules (Fig. 5). Taken altogether, hydrophobicity is a quantitative predictor to evaluate the toxic effect of a metabolite on microbial health.

## Conclusions

Analysis of a comprehensive list of short-chain alcohols, acids, and esters shows distinctive toxic effects of these metabolites on *E. coli* health. Alcohols are most toxic followed by acids and esters at identical concentrations and total carbon counts. Regardless of metabolite classes and concentrations, longer chain metabolites inhibit microbial growth more than the shorter chain ones. Branched chain metabolites are less toxic than straight chain ones with the same total carbon count. Remarkably, for the same total carbon counts, esters having longer chain alcohol moieties are more inhibitory than those having short-chain alcohol moieties. Hydrophobicity of a metabolite is a good quantitative index to determine its toxic effect on microbial health. Since this study focuses on characterizing the toxic effects of fermentative metabolites on an industrial workhorse gram-negative bacterium *E. coli*, it is of particular interest to further explore in the future whether the trends found in this study exist in other bacterial, eukaryotic, and archaeal species. Although it is not the focus of the study, fermentative metabolites can cause cytotoxicity when they are present inside the cells beyond membrane damage [23, 24, 49]. Overall, this study sheds light on the toxic effects of fermentative metabolites with distinct characteristics on microbial growth and helps in the selection of desirable metabolites and hosts for industrial fermentation to overproduce them.

## Methods

### Medium and cell culturing

For all *E. coli* MG1655 (DE3) characterization experiments, modified M9 medium (pH ~ 7) was used, consisting of 100 mL/L of 10X M9 salts, 1 mL/L of 1 M MgSO_4_, 100 μL/L of 1 M CaCl_2_, 1 mL/L of stock thiamine HCl solution (1 g/L), 1 ml/L of stock trace metal solution, 10 g/L glucose, and 5 g/L yeast extract [50]. 10X M9 salts are composed of 70 g/L Na_2_HPO_4_·H_2_O, 30 g/L KH_2_PO_4_, 5 g/L NaCl, and 10 g/L NH_4_Cl. Alcohols, esters, and acids were added at necessary concentrations into flasks of partitioned media. Media with the chemicals of interest were then transferred from the flasks to 28-mL Balch tubes and capped with rubber stoppers and aluminum seals to create anaerobic environment. In cases where chemical solubility prevented making well-mixed stocks of media and compounds, each chemical was added via a Hamilton glass syringe to the tube described above. After the addition of each chemical, media were pH adjusted to 7 with 5 M KOH. Alcohols, acids, and esters were studied at varying concentrations based on a combination of factors including solubility and observed toxicity.

Stock cells from the − 80 °C freezer were streaked onto lysogeny broth (LB)-agar plates and then were grown overnight in flasks containing 50 mL of the modified M9 medium in a New Brunswick Excella E25 incubator at 37 °C and 175 rpm until OD_600nm_ (optical density measured at 600 nm using a Thermo Scientific Genesys 30 Visible Spectrophotometer) reached 2.5–3.0. In the event that this OD setpoint was surpassed, cells were diluted in 50 mL of the same medium to OD = 1.0 and grown once again to OD = 2.5. Cells were transferred to nitrogen-sparged, anaerobic culture Balch tubes containing 20 mL of media at initial OD = 0.1 to begin growth characterization on a 75° angled platform under identical conditions. Cell culture densities were measured throughout 24-h fermentation. All experiments were performed in at least 3 biological replicates.

### Data collection and analysis

#### Partition coefficient

Partition coefficient, a measure of hydrophobicity of a metabolite, was calculated as follows:

1 $${ \log }_{10} P_{i} = { \log }_{10} \left( {\frac{{{\text{S}}_{\text{i}}^{\text{octanol}} }}{{{\text{S}}_{\text{i}}^{\text{water}} }}} \right),$$

where *P*_*i*_ is the partition coefficient of metabolite *i* and *S*_*i*_^octanol^ and *S*_*i*_^water^ (g/L) are the solubilities of metabolite *i* in octanol and water, respectively. *P*_*i*_ was calculated at room temperature and atmospheric pressure using the Molinspiration Cheminformatics interactive log(P) calculator [51]. The input for this calculator used the SMILES chemical notation acquired from PubChem [52].

#### ONMED

Octane Normalized Mass Energy Density (ONMED) was calculated as the ratio of standard heat of combustion of a metabolite to that of octane (~ 44.5 kJ/kg) [18] where the standard heat of combustion of each chemical was estimated based on average bond energies [53].

#### Polarity

The polarity of molecules, in debyes (D), was estimated using MolCalc [54], a web application for estimating physiochemical properties of a molecule.

#### Total surface area

The total surface area of a molecule (A^2^, where A is the Angstrom unit, 1A = 10^−10^ m) was calculated using MolCalc [54].

#### Specific growth rate

First-order kinetics was applied to calculate a specific growth rate from kinetic measurement of cell growth as follows:

2 $$\mu = \frac{1}{{{\text{C}}_{X} }} \cdot \frac{{{\text{dC}}_{X} }}{\text{dt}},$$

where *μ* (1/h) is the specific growth rate, C_*X*_ (g/L) is the cell titer, and *t* (h) is the culturing time. Note that in our study cell titer was estimated from the measured OD with a correlation of 1 OD ~ 0.5 g DCW/L.

## Additional files

**Additional file 1.** Additional figures and descriptions in a PDF.

**Additional file 2.** Raw data of strain characterization and physical properties of metabolites.

## Abbreviations

μ: specific growth rate
C_X_: cell concentration
DCW: dry cell weight
OD: optical density
ONMED: octane normalized mass energy density
P_i_: partition coefficient of metabolite i
S_i_^octanol^ and S_i_^water^: solubilities of metabolite i in octanol and water, respectively
t: time
h: hour
D: debyes
A: angstrom

## References

[1] Smith PM, Howitt MR, Panikov N, Michaud M, Gallini CA, Bohlooly YM, Glickman JN, Garrett WS (2013). The microbial metabolites, short-chain fatty acids, regulate colonic Treg cell homeostasis. Science.

[2] Agler MT, Wrenn BA, Zinder SH, Angenent LT (2011). Waste to bioproduct conversion with undefined mixed cultures: the carboxylate platform. Trends Biotechnol.

[3] Ingram L, Conway T, Clark D, Sewell G, Preston J (1987). Genetic engineering of ethanol production in *Escherichia coli*. Appl Environ Microbiol.

[4] Trinh C, Srienc F (2009). Metabolic engineering of *Escherichia coli* for efficient conversion of glycerol to ethanol. Appl Environ Microbiol.

[5] Hanai T, Atsumi S, Liao J (2007). Engineered synthetic pathway for isopropanol production in *Escherichia coli*. Appl Environ Microbiol.

[6] Inui M (2008). Expression of *Clostridium acetobutylicum* butanol synthetic genes in *Escherichia coli*. Appl Microbiol Biotechnol.

[7] Atsumi S, Hanai T, Liao JC (2008). Non-fermentative pathways for synthesis of branched-chain higher alcohols as biofuels. Nature.

[8] Tseng H-C, Prather KL (2012). Controlled biosynthesis of odd-chain fuels and chemicals via engineered modular metabolic pathways. Proc Natl Acad Sci.

[9] Dekishima Y, Lan EI, Shen CR, Cho KM, Liao JC (2011). Extending carbon chain length of 1-butanol pathway for 1-hexanol synthesis from glucose by engineered *Escherichia coli*. J Am Chem Soc.

[10] Nakamura CE, Whited GM (2003). Metabolic engineering for the microbial production of 1,3-propanediol. Curr Opin Biotechnol.

[11] Yim H, Haselbeck R, Niu W, Pujol-Baxley C, Burgard A, Boldt J, Khandurina J, Trawick JD, Osterhout RE, Stephen R (2011). Metabolic engineering of *Escherichia coli* for direct production of 1, 4-butanediol. Nat Chem Biol.

[12] Causey T, Shanmugam K, Yomano L, Ingram L (2004). Engineering *Escherichia coli* for efficient conversion of glucose to pyruvate. Proc Natl Acad Sci USA.

[13] Chang D-E, Jung H-C, Rhee J-S, Pan J-G (1999). Homofermentative production of d-orl-lactate in metabolically engineered *Escherichia coli* RR1. Appl Environ Microbiol.

[14] Torella JP, Ford TJ, Kim SN, Chen AM, Way JC, Silver PA (2013). Tailored fatty acid synthesis via dynamic control of fatty acid elongation. Proc Natl Acad Sci.

[15] Sánchez AM, Bennett GN, San K-Y (2005). Novel pathway engineering design of the anaerobic central metabolic pathway in *Escherichia coli* to increase succinate yield and productivity. Metab Eng.

[16] Yu JL, Xia XX, Zhong JJ, Qian ZG (2014). Direct biosynthesis of adipic acid from a synthetic pathway in recombinant *Escherichia coli*. Biotechnol Bioeng.

[17] Rodriguez GM, Tashiro Y, Atsumi S (2014). Expanding ester biosynthesis in *Escherichia coli*. Nat Chem Biol.

[18] Layton DS, Trinh CT (2016). Microbial synthesis of a branched-chain ester platform from organic waste carboxylates. Metab Eng Commun.

[19] Layton DS, Trinh CT (2016). Expanding the modular ester fermentative pathways for combinatorial biosynthesis of esters from volatile organic acids. Biotechnol Bioeng..

[20] Layton DS, Trinh CT (2014). Engineering modular ester fermentative pathways in *Escherichia coli*. Metab Eng.

[21] Warnecke T, Gill RT (2005). Organic acid toxicity, tolerance, and production in *Escherichia coli* biorefining applications. Microb Cell Fact.

[22] Jarboe LR, Royce LA, Liu P (2013). Understanding biocatalyst inhibition by carboxylic acids. Front Microbiol.

[23] Brynildsen MP, Liao JC (2009). An integrated network approach identifies the isobutanol response network of *Escherichia coli*. Mol Syst Biol.

[24] Atsumi S, Wu TY, Machado IM, Huang WC, Chen PY, Pellegrini M, Liao JC (2010). Evolution, genomic analysis, and reconstruction of isobutanol tolerance in *Escherichia coli*. Mol Syst Biol.

[25] Huffer S, Clark ME, Ning JC, Blanch HW, Clark DS (2011). Role of alcohols in growth, lipid composition, and membrane fluidity of yeasts, bacteria, and archaea. Appl Environ Microbiol.

[26] Lennen RM, Kruziki MA, Kumar K, Zinkel RA, Burnum KE, Lipton MS, Hoover SW, Ranatunga DR, Wittkopp TM, Marner WD (2011). Membrane stresses induced by overproduction of free fatty acids in *Escherichia coli*. Appl Environ Microbiol.

[27] Royce LA, Liu P, Stebbins MJ, Hanson BC, Jarboe LR (2013). The damaging effects of short chain fatty acids on *Escherichia coli* membranes. Appl Microbiol Biotechnol.

[28] Zaldivar J, Ingram LO (1999). Effect of organic acids on the growth and fermentation of ethanologenic *Escherichia coli* LY01. Biotechnol Bioeng.

[29] Nicolaou SA, Gaida SM, Papoutsakis ET (2010). A comparative view of metabolite and substrate stress and tolerance in microbial bioprocessing: from biofuels and chemicals, to biocatalysis and bioremediation. Metab Eng.

[30] Lennen RM, Pfleger BF (2013). Modulating membrane composition alters free fatty acid tolerance in *Escherichia coli*. PLoS ONE.

[31] Sherkhanov S, Korman TP, Bowie JU (2014). Improving the tolerance of *Escherichia coli* to medium-chain fatty acid production. Metab Eng.

[32] Tan Z, Yoon JM, Nielsen DR, Shanks JV, Jarboe LR (2016). Membrane engineering via trans unsaturated fatty acids production improves *Escherichia coli* robustness and production of biorenewables. Metab Eng.

[33] Dunlop MJ, Dossani ZY, Szmidt HL, Chu HC, Lee TS, Keasling JD, Hadi MZ, Mukhopadhyay A (2011). Engineering microbial biofuel tolerance and export using efflux pumps. Mol Syst Biol.

[34] Zingaro KA, Papoutsakis ET (2013). GroESL overexpression imparts Escherichia coli tolerance to i-, n-, and 2-butanol, 1, 2, 4-butanetriol and ethanol with complex and unpredictable patterns. Metab Eng.

[35] Trček J, Mira NP, Jarboe LR (2015). Adaptation and tolerance of bacteria against acetic acid. Appl Microbiol Biotechnol.

[36] Woodruff LB, Boyle NR, Gill RT (2013). Engineering improved ethanol production in *Escherichia coli* with a genome-wide approach. Metab Eng.

[37] Sandoval NR, Kim JY, Glebes TY, Reeder PJ, Aucoin HR, Warner JR, Gill RT (2012). Strategy for directing combinatorial genome engineering in *Escherichia coli*. Proc Natl Acad Sci.

[38] Zaldivar J, Martinez A, Ingram LO (2000). Effect of alcohol compounds found in hemicellulose hydrolysate on the growth and fermentation of ethanologenic *Escherichia coli*. Biotechnol Bioeng.

[39] Knoshaug EP, Zhang M (2009). Butanol tolerance in a selection of microorganisms. Appl Biochem Biotechnol.

[40] Alakomi H-L, Skyttä E, Saarela M, Mattila-Sandholm T, Latva-Kala K, Helander I (2000). Lactic acid permeabilizes gram-negative bacteria by disrupting the outer membrane. Appl Environ Microbiol.

[41] DiRienzo JM, Inouye M (1979). Lipid fluidity-dependent biosynthesis and assembly of the outer membrane proteins of *E. coli*. Cell.

[42] Cho C-W, Pham TPT, Jeon Y-C, Vijayaraghavan K, Choe W-S, Yun Y-S (2007). Toxicity of imidazolium salt with anion bromide to a phytoplankton *Selenastrum capricornutum*: effect of alkyl-chain length. Chemosphere.

[43] Garcia M, Campos E, Sanchez-Leal J, Ribosa I (1999). Effect of the alkyl chain length on the anaerobic biodegradability and toxicity of quaternary ammonium based surfactants. Chemosphere.

[44] Dombek KM, Ingram L (1984). Effects of ethanol on the *Escherichia coli* plasma membrane. J Bacteriol.

[45] Ingólfsson HI, Andersen OS (2011). Alcohol’s effects on lipid bilayer properties. Biophys J.

[46] Wong P, Chau Y, Kramar O, Bengert G (1982). Structure–toxicity relationship of tin compounds on algae. Can J Fish Aquat Sci.

[47] Sikkema J, De Bont J, Poolman B (1994). Interactions of cyclic hydrocarbons with biological membranes. J Biol Chem.

[48] Sikkema J, De Bont J, Poolman B (1995). Mechanisms of membrane toxicity of hydrocarbons. Microbiol Rev.

[49] Royce LA, Boggess E, Fu Y, Liu P, Shanks JV, Dickerson J, Jarboe LR (2014). Transcriptomic analysis of carboxylic acid challenge in *Escherichia coli*: beyond membrane damage. PLoS ONE.

[50] Trinh CT, Li J, Blanch HW, Clark DS (2011). Redesigning *Escherichia coli* metabolism for anaerobic production of isobutanol. Appl Environ Microbiol.

[51] Mannhold R, Poda GI, Ostermann C, Tetko IV (2009). Calculation of molecular lipophilicity: state-of-the-art and comparison of log P methods on more than 96,000 compounds. J Pharm Sci.

[52] Acland A, Agarwala R, Barrett T, Beck J, Benson DA, Bollin C, Bolton E, Bryant SH, Canese K, Church DM (2014). Database resources of the national center for biotechnology information. Nucleic Acids Res.

[53] Zumdahl SS, Susan A (2000). Chemistry.

[54] Jensen JH, Kromann JC (2013). The molecule calculator: a web application for fast quantum mechanics-based estimation of molecular properties.

