# Supplementary material for: Comprehensive characterization of toxicity of fermentative metabolites on microbial growth
Source: Biotechnol Biofuels. 2017 Nov 30;10:262. doi: 10.1186/s13068-017-0952-4 (PMC5707818; doi:10.1186/s13068-017-0952-4)

Additional File 1

Comprehensive Characterization of Toxicity of Fermentative Metabolites on Microbial Growth

Brandon Wilbanks[1] and Cong T. Trinh[1,2,*]

[1]Department of Chemical and Biomolecular Engineering, University of Tennessee, Knoxville, TN 37920

[2]Bioenergy Science Center (BESC), Oak Ridge National Laboratory, Oak Ridge

[*]Corresponding author:

Cong T. Trinh

Dept of Chemical and Biomolecular Engineering

University of Tennessee, Knoxville

1512 Middle Dr., DO#432

Knoxville, TN 37996

ctrinh@utk.edu

Additional Contact Information:

Brandon Wilbanks: brandonawilbanks@gmail.com

Figure S1: Growth kinetics of *E. coli* exposed to eight short-chain alcohols at various concentrations including **(A)** ethanol, **(B)** propanol, **(C)** isopropanol, **(D)** butanol, **(E)** isobutanol, **(F)** pentanol, **(G)** isopentanol, and **(H)** hexanol

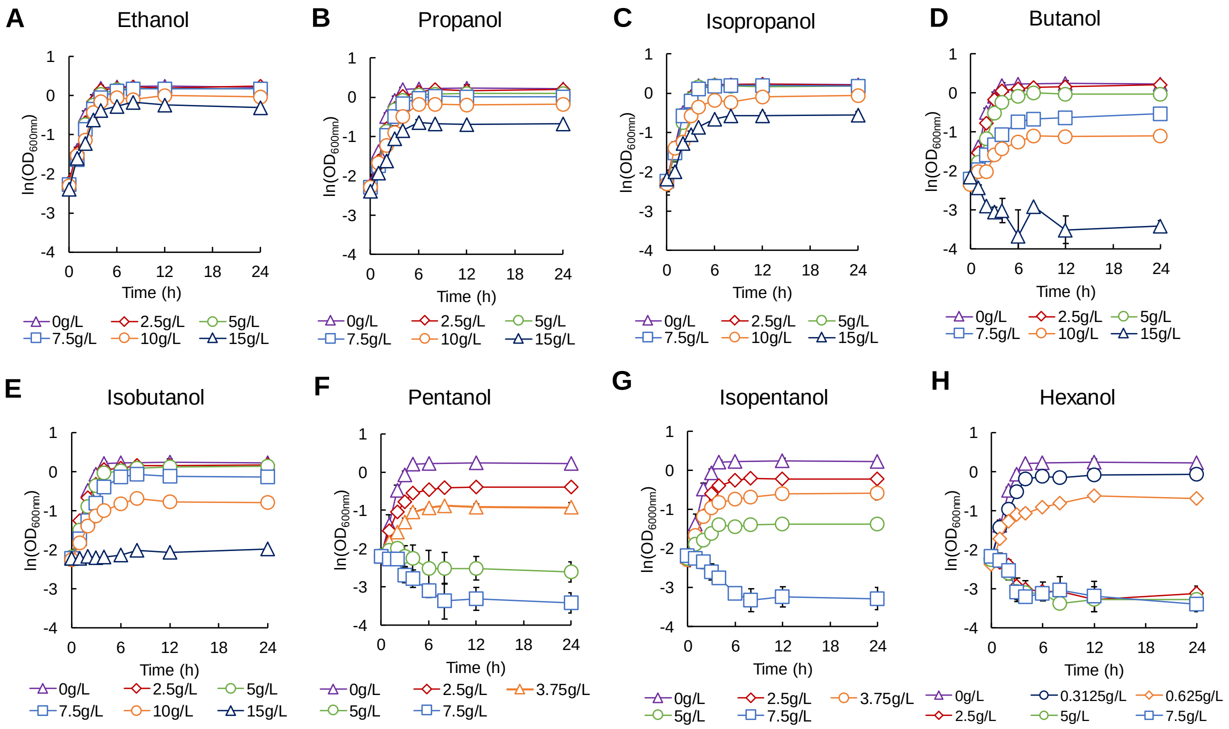

Figure S2: Growth kinetics of *E. coli* exposed to eight short-chain fatty acids at various concentrations including **(A)** acetic acid, **(B)** propanoic acid, **(C)** butanoic acid, **(D)** isobutanoic acid, **(E)** pentanoic acid, **(F)** isopentanoic acid, **(G)** hexanoic aid, and **(H)** octanoic acid

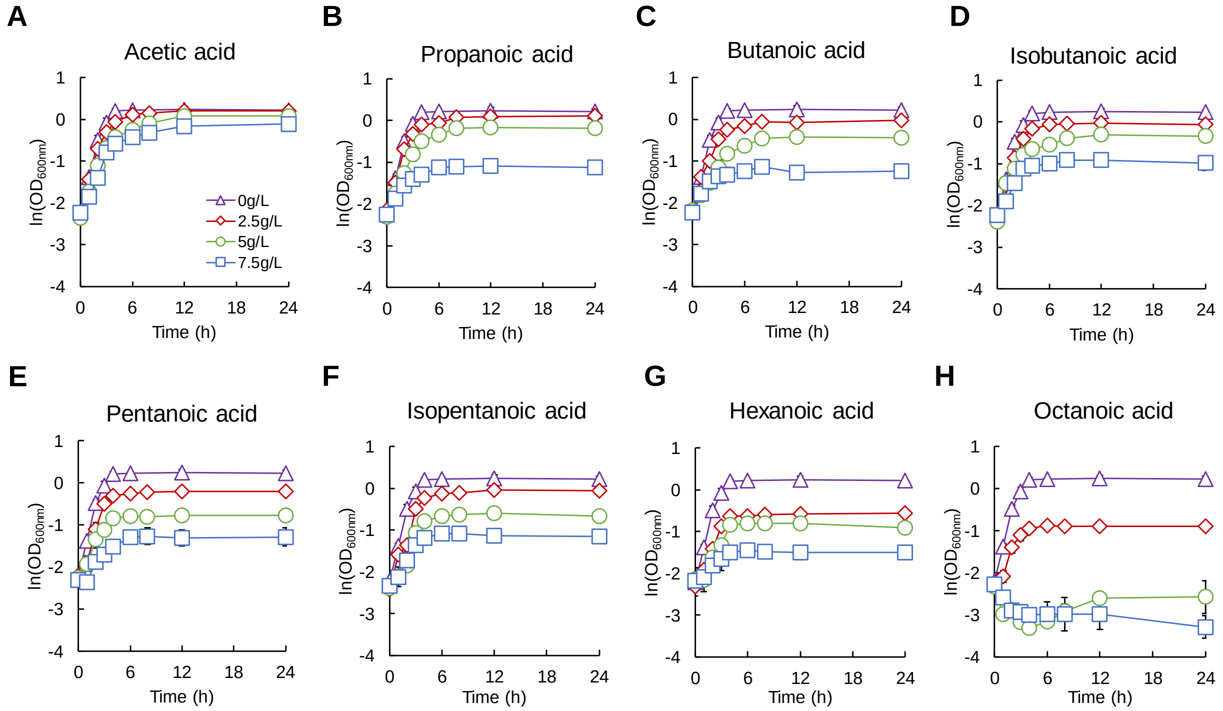

Figure S3: Growth kinetics of *E. coli* exposed to sixteen short-chain esters at various concentrations including **(A-D)** ethyl esters, **(E-J)** (iso)propyl esters, and **(K-P)** (iso)butyl esters

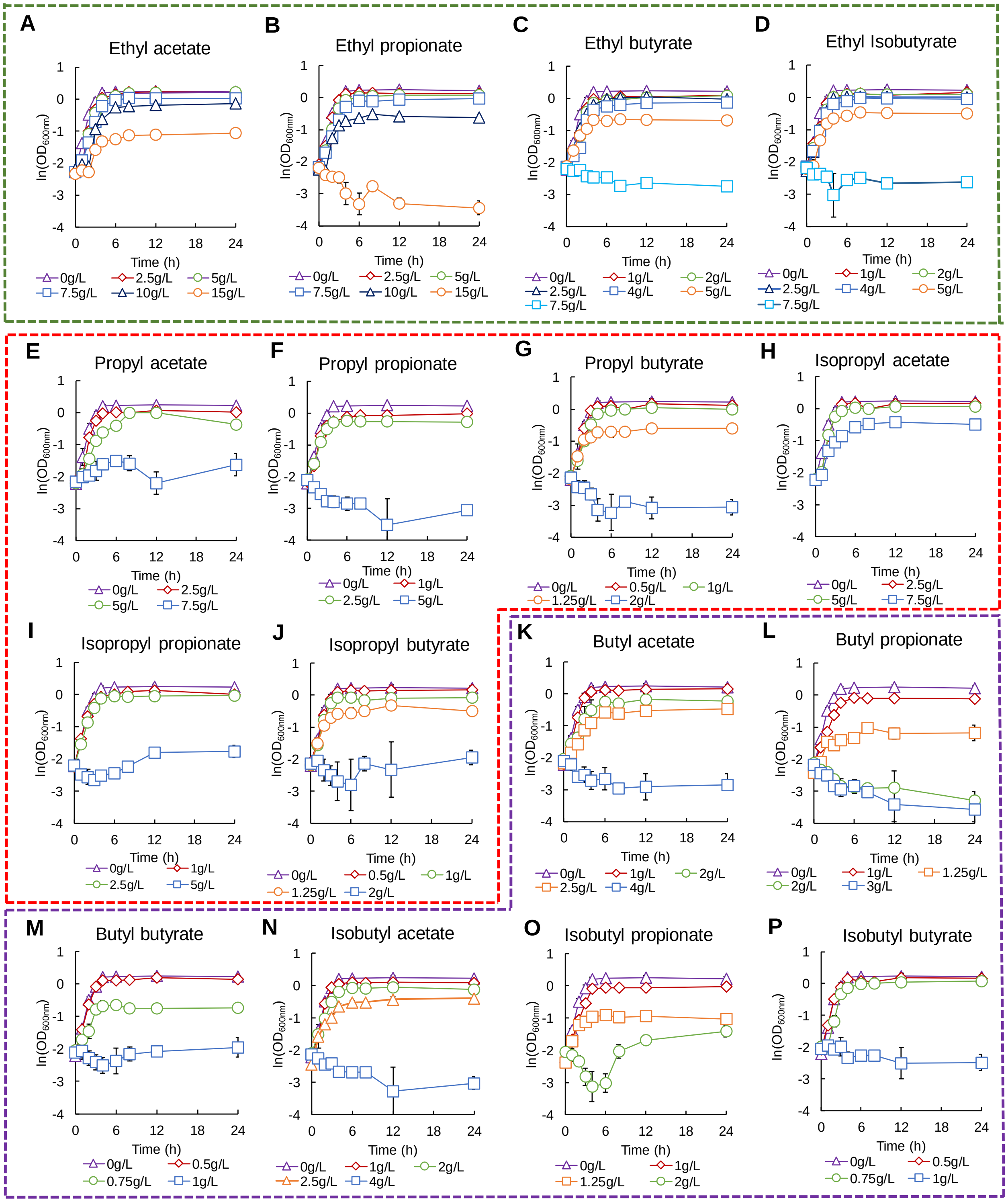

Figure S4: Correlation between growth rate and maximum OD of *E. coli* exposed to alcohols, acids, and esters at various concentrations

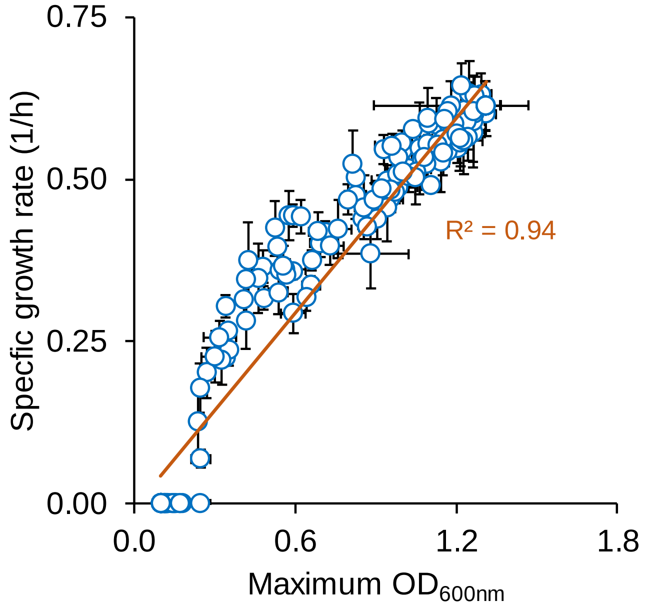

Figure S5: Effects of carbon numbers of alcohols on **(A-C)** specific growth rate and **(D-F)** maximum OD. Filled symbol: straight chain, open symbol: branched chain

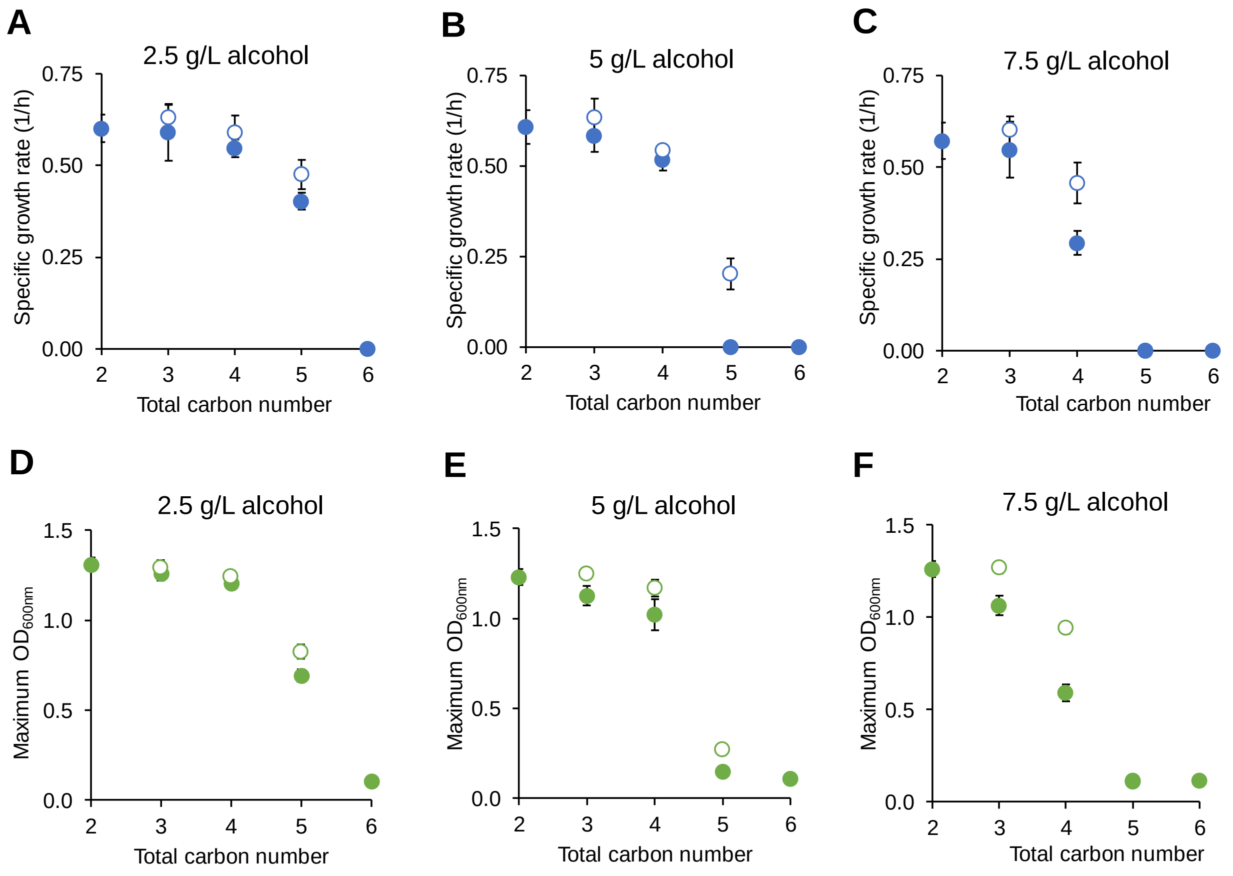

Figure S6: Effects of carbon numbers of acids on **(A-C)** specific growth rate and **(D-F)** maximum OD. Filled symbol: straight chain, open symbol: branched chain

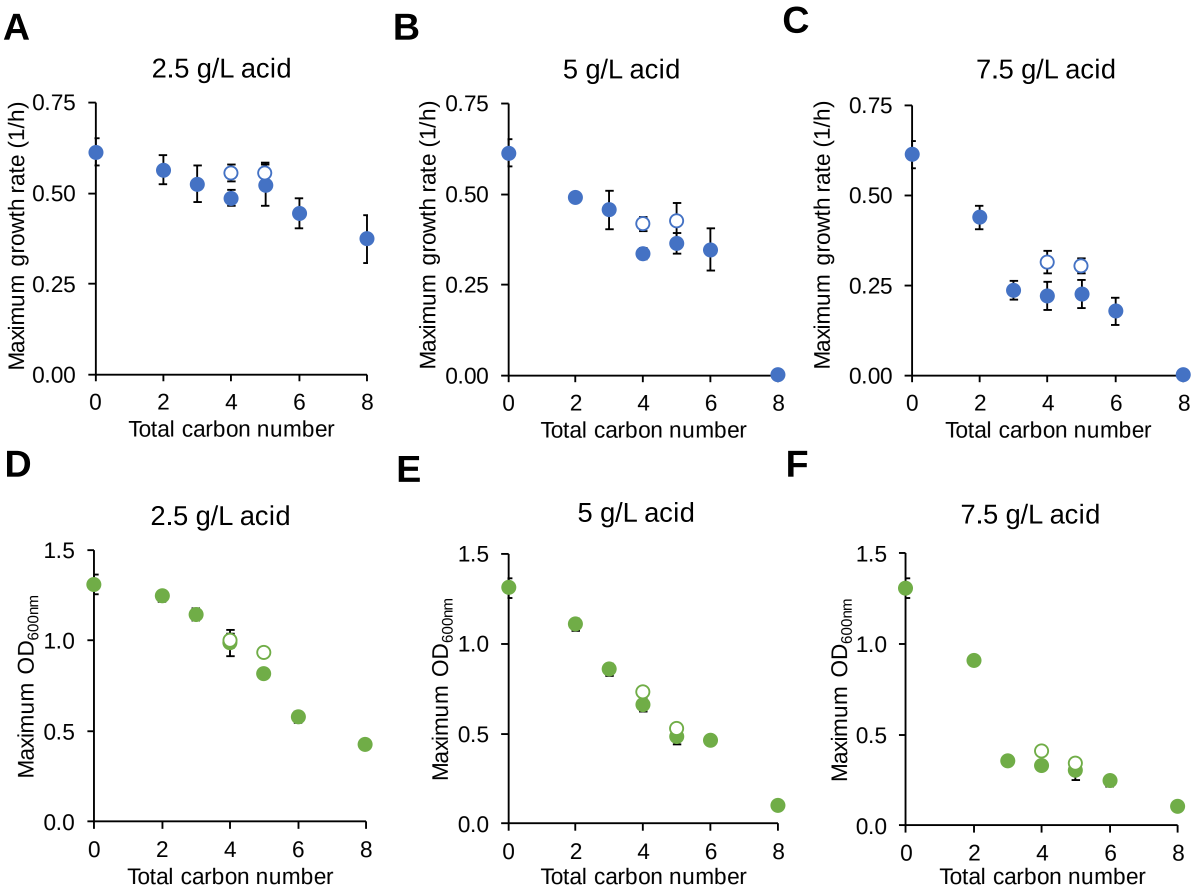

Figure S7: Effects of carbon numbers of alcohols on **(A-C)** specific growth rate and **(D-F)** maximum OD. Filled symbol: straight chain, open symbol: branched chain

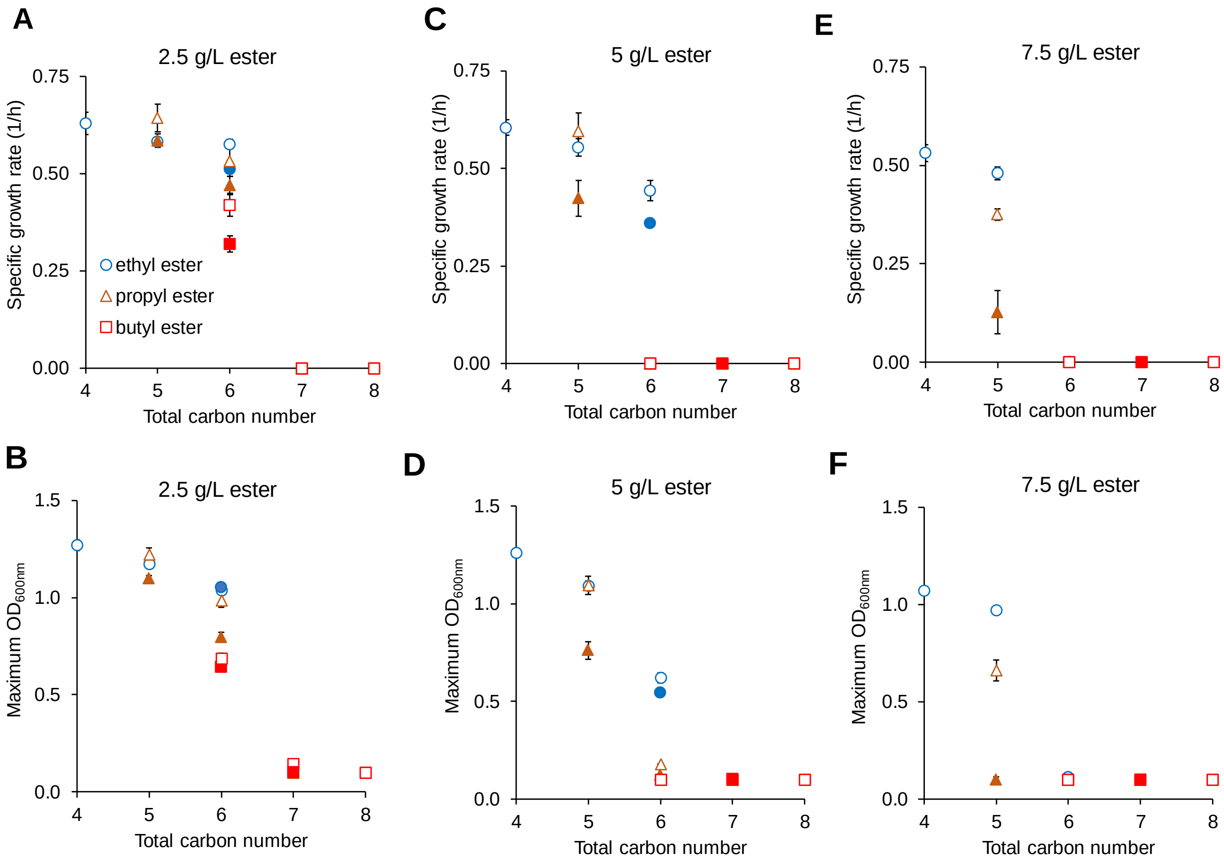

Supplement: Supplementary file 1 — Additional file 1. Additional figures and descriptions in a PDF. [file 13068_2017_952_MOESM1_ESM.pdf]
